# Correcting Calcium Dysregulation in Chronic Heart Failure Using *SERCA2a* Gene Therapy

**DOI:** 10.3390/ijms19041086

**Published:** 2018-04-05

**Authors:** T. Jake Samuel, Ryan P. Rosenberry, Seungyong Lee, Zui Pan

**Affiliations:** 1 Department of Kinesiology, College of Nursing and Health Innovation, The University of Texas at Arlington, Arlington, TX 76019, USA; Thomas.Samuel@uta.edu (T.J.S.); ryan.rosenberry@uta.edu (R.P.R.); seungyong.lee@uta.edu (S.L.); 2 Department of Graduate Nursing, College of Nursing and Health Innovation, The University of Texas at Arlington, Arlington, TX 76019, USA

**Keywords:** calcium handling, SERCA2a, chronic heart failure, clinical trial, adeno-associated virus (AAV), gene delivery

## Abstract

Chronic heart failure (CHF) is a major contributor to cardiovascular disease and is the leading cause of hospitalization for those over the age of 65, which is estimated to account for close to seventy billion dollars in healthcare costs by 2030 in the US alone. The successful therapies for preventing and reversing CHF progression are urgently required. One strategy under active investigation is to restore dysregulated myocardial calcium (Ca^2+^), a hallmark of CHF. It is well established that intracellular Ca^2+^ concentrations are tightly regulated to control efficient myocardial systolic contraction and diastolic relaxation. Among the many cell surface proteins and intracellular organelles that act as the warp and woof of the regulatory network controlling intracellular Ca^2+^ signals in cardiomyocytes, sarco/endoplasmic reticulum Ca^2+^ ATPase type 2a (SERCA2a) undoubtedly plays a central role. SERCA2a is responsible for sequestrating cytosolic Ca^2+^ back into the sarcoplasmic reticulum during diastole, allowing for efficient uncoupling of actin-myosin and subsequent ventricular relaxation. Accumulating evidence has demonstrated that the expression of SERCA2a is downregulated in CHF, which subsequently contributes to severe systolic and diastolic dysfunction. Therefore, restoring SERCA2a expression and improving cardiomyocyte Ca^2+^ handling provides an excellent alternative to currently used transplantation and mechanical assist devices in the treatment of CHF. Indeed, advancements in safe and effective gene delivery techniques have led to the emergence of *SERCA2a* gene therapy as a potential therapeutic choice for CHF patients. This mini-review will succinctly detail the progression of *SERCA2a* gene therapy from its inception in plasmid and animal models, to its clinical trials in CHF patients, highlighting potential avenues for future work along the way.

## 1. Introduction

Chronic heart failure (CHF) is a major form of cardiovascular disease and is the leading cause of hospitalization for those over the age of 65 [1]. It is expected to account for almost seventy billion dollars in healthcare costs by 2030 in the US alone [2]. The current pharmacological therapies for CHF merely target symptom management, with limited success in treating the underlying etiology of the disease. The late stage CHF patients eventually need expensive and hard-to-obtain heart transplantation or mechanical assist devices. Therefore, successful therapies for preventing and reversing CHF progression are urgently required.

**Figure 1:**
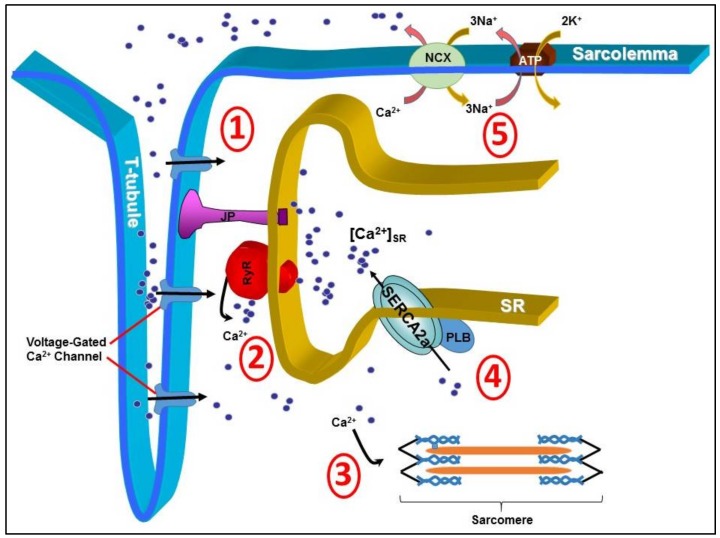
Cardiac intracellular Ca^2+^ is tightly regulated by several proteins. Efficient systolic contraction and diastolic relaxation is reliant on efficient Ca^2+^ handling through 5 main processes. (1) Diffusion of Ca^2+^ in to the cytosol via voltage-gated Ca^2+^ channels (DHPRs) located on the surface of the transverse-tubule (T-tubule); (2) Ca^2+^-induced-Ca^2+^-release from the ryanodine receptors (RyRs); (3) Binding of Ca^2+^ to troponin-C in the sarcomere, stimulating actin-myosin cross-linking; (4) Sequestration of Ca^2+^ back in to the sarcoplasmic reticulum via the important Ca^2+^ pump sarco/endoplasmic reticulum Ca^2+^ ATPase (SERCA2a); and (5) Expulsion of Ca^2+^ from the cell via sodium-calcium exchanger pumps (NCX). JP, junctophilins; PLB, phopholamban; ATP, ATP pump; 3Na^+^, sodium; K^+^, potassium.

Understanding the basic mechanisms involved in the development of CHF has been an active field of research in the quest to identify abnormalities that could potentially be targeted by gene transfer. It has been well established that myocardial calcium (Ca^2+^) dysregulation is a hallmark of CHF. Tight control of cardiac intracellular Ca^2+^ handling plays an integral role in synchronous actin-myosin cross-bridge cycling and the resulting systolic contraction and diastolic relaxation. Many proteins either at the cell surface or intracellular organelles form the warp and woof of the regulatory network for intracellular Ca^2+^ signals in cardiomyocytes (Figure 1). During systole, Ca^2+^ enters the sarcolemma through L-type Ca^2+^ channels, i.e., dihydropyrodine receptor (DHPR) located on the transverse-tubules (T-tubules), before diffusing across the short distance to the ryanodine receptors (RyRs) located to the sarcoplasmic reticulum (SR). This small amount of Ca^2+^ influx triggers RyRs to generate further Ca^2+^-induced-Ca^2+^-release (CICR) from the SR to form magnified Ca^2+^ transient [3]. Ca^2+^ then binds to troponin-C in the sarcomere and stimulates actin-myosin cross-bridge linking, i.e., systolic contraction [4]. During diastole, in order to reduce the likelihood of Ca^2+^ binding to troponin-C and causing prolonged myocardial systolic contraction, Ca^2+^ is actively sequestrated back into the SR via a sarco/endoplasmic reticulum Ca^2+^ ATPase, SERCA2a in cardiomyocyte [5,6], or expelled from the sarcolemma by sodium-Ca^2+^ exchanger pumps (NCX) [3]. As such, SERCA2a is undoubtedly essential for complete myocyte Ca^2+^ homeostasis.

Dysregulation of any of the above Ca^2+^ handling processes will result in impaired ventricular contractility and impaired myocyte relaxation, leading to cardiac dysfunction [4,7,8]. While efforts have been devoted to investigating the therapeutic value of many Ca^2+^ channels and proteins in regulating cytosolic Ca^2+^ concentrations, SERCA2a has received the most interest in recent years.

**Figure 2:**
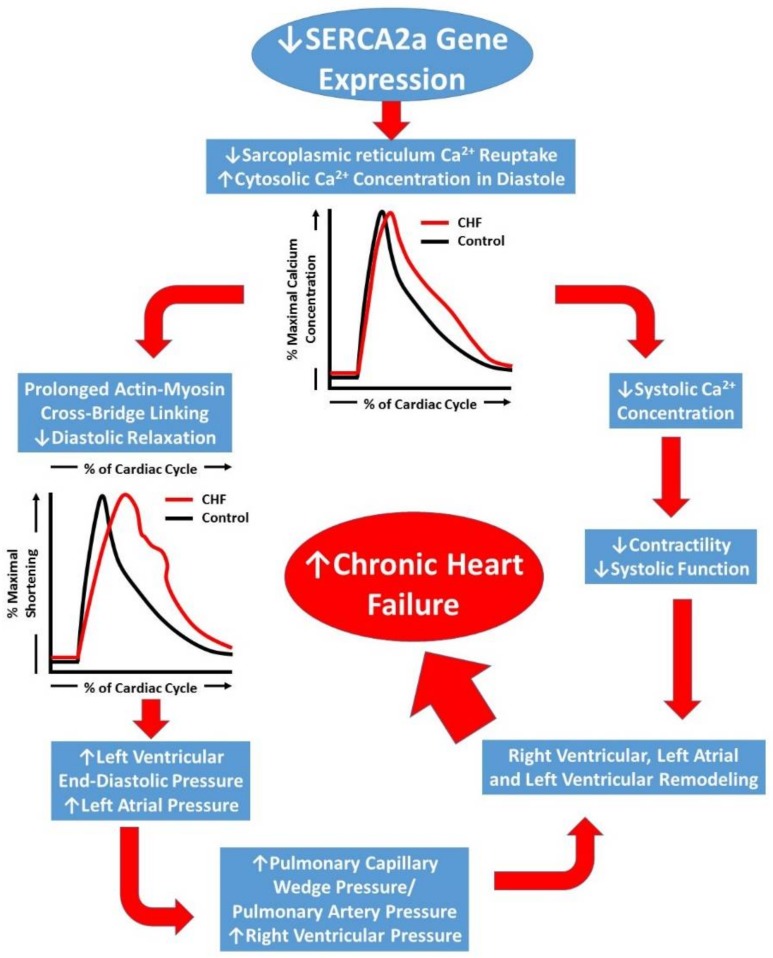
Schematic representation of the progression of chronic heart failure. Initial downregulation of SERCA2a function leads to increased cytosolic Ca^2+^, which ultimately compromises left ventricular contractility (systolic function) and leads to prolonged left ventricular relaxation. Prolonged relaxation leads to increased filling pressures and a backlog of pressure in to the pulmonary circulation and right heart, the result of which leads to severe right and left heart remodeling and chronic heart failure development.

Reduction in SERCA2a levels in the SR has been reported in failing heart tissues [9,10]. Impaired Ca^2+^-ATPase pump function and decreased *SERCA2a* gene transcription has recently been attributed to delayed left ventricular relaxation and increased left ventricular filling pressures [11] which may lead to pulmonary edema, a common symptom in CHF (Figure 2). Biotechnological advances over the past two decades have led to gene therapy becoming a viable option for treating many pathologic conditions—including CHF [12,13,14]. Matter of fact, SERCA2a had been identified as a possible gene therapy target as early as 1978 [15]. This review will focus on *SERCA2a* gene therapy in CHF and attempt to highlight the seminal investigations using early gene delivery techniques in animals that contributed to the early promise for its use in humans. It will also provide a summary of the major human clinical trials which identified several limitations to these methods for treating human CHF. Finally, recent novel developments in gene delivery and targeting will be discussed and future directions for this field of research will be proposed.

## 2. Early Techniques of *SERCA2a* Gene Manipulation in Animals

The role of cytosolic Ca^2+^ in cardiac myocyte contraction has been intensely studied for decades. As far back as 40 years ago, investigators were purifying SERCA proteins to better understand the molecular mechanisms through which Ca^2+^ is transported from the cytosol into the sarcoplasmic reticulum (SR) [15]. Shortly following the identification of Ca^2+^ channels as key mediators of cardiac function, they became therapeutic targets in the treatment of various cardiac ailments [16,17,18]. With the rise of interest in genomics and gene editing techniques, the direct manipulation of Ca^2+^ channels or pumps, and thus manipulation of Ca^2+^ handling in cardiac myocytes, became possible.

### 2.1. Plasmid and Direct DNA Injection

The earliest successful approaches of manipulating *SERCA2a* gene expression in animal models were performed using direct insertion of plasmids into developing mouse oocytes. The rat SERCA2a cDNA was cloned into plasmids containing mouse cardiac α-MHC promoter. While certain repetitive and unnecessary exons were removed, an additional human growth hormone polyadenylation site was included to promote both polyadenylation and termination [9]. Once these sequences are successfully cloned into the plasmid, restriction endonucleases are applied to cleave the gene from the plasmid. The linear DNA fragment can then be purified for direct injection into oocytes. The resulting transgenic mice possessed extra copies of the *SERCA2a* gene and exhibited increased expression of the protein [9,19,20].

Initial studies applying this transgenic technique sought to characterize and measure the effects of the increased SERCA2a expression in otherwise unaltered mice. Early investigations found that overexpression of the *SERCA2a* gene in a transgenic mouse model resulted in improved Ca^2+^ handling which translated to augmented myocyte contraction and relaxation [9,10,20]. Of note, the transgenic mice had a significantly shorter relaxation time indicating improved relaxation kinetics, consistent with improved Ca^2+^ sequestration via SERCA2a [20]. With these positive proof-of-concept results, investigators next sought to determine whether SERCA2a could improve cardiac functionality in disease stages. To test this potential treatment, an aortic stenosis-induced CHF model was employed in transgenic mice. The increased afterload from the artificial stenosis caused left ventricular hypertrophy, mimicking the conditions of early CHF. In this investigation, the transgenic expression of SERCA2a provided chronic protective effects against the progression of CHF [19].

Although these early studies demonstrated positive and profound effects from direct injection of DNA fragment containing *SERCA2a* gene, there are substantial limitations inherent to this technique. First, this laboratory procedure requires a significant time commitment and many uncontrollable steps before the success of the transfection can be determined. In the case of He et al. super-ovulated eggs were treated with purified SERCA2a DNA, gestated for 20 days, and the newborn mice were allowed to develop for three weeks before testing tail samples for SERCA2a expression by Southern blotting [20]. This process relies on integration of the gene into the embryonic genome, normal gestation and delivery, and inerrant expression in the developed animal. Even if each step is performed to perfection, the imprecision introduces excessive error and contributes to study failure. In fact, only three mice out of nine integrated the SERCA2a plasmid. Of these three mice, the single male was sterile, leaving only two female transgenic mice that successfully passed the gene on to their offspring [20].

A second limitation of this technique is that for additional copies of SERCA2a to be expressed, all biological machinery in the transcription/translation/post-translational modification pathway must also function in a normal and healthy manner. Direct injection of the DNA into oocytes carries the potential risk for improper integration of the gene into the genome. As evidenced by the low number of successfully reared transgenic mice, the possibility for lethality or sterility is considerable. Furthermore, this approach cannot be applied to developed organisms, hindering its expansion and/or adaptation into other animal models. To further investigate SERCA2a as an intervention, it was necessary to explore more advanced and versatile gene modifying techniques.

Last and foremost, direct manipulation of human oocytes raises serious ethical issues, which disqualify this approach from developing into a useful human gene therapy for CHF. Rather, these transgenic animal studies laid the theoretical understanding that prompted improvements in biotechnological techniques to tackle this problem. Alternative ethical and safer approaches have been called upon.

### 2.2. Adenoviral (Ad)-Based Vectors

Ad are double-strand DNA vectors that bind to and enter the cell membrane before being transported to the nucleus, allowing transfection of genes into a variety of cells such as cardiomyocytes, skeletal muscle and smooth muscle myocytes [21]. Delivery of Ad-based vectors to cardiomyocytes through intracoronary infusion and direct myocardial injection has showed some success in transduction of the target gene [22]. Although Ad-based vectors attain a high level of cellular transduction, the various Ad serotypes result in varied efficiency [21], leading to mixed success in modulating target gene expression.

Ad mediated *SERCA2a* gene delivery (Ad/SERCA2a) was reported to present improvements in cardiac function in CHF due to the upregulation of *SERCA2a* genes. Ad/*SERCA2a* gene transfer in mice with CHF enhances contractile function [23] and restores phosphocreatine and ATP level in the heart, which represents the recovery of heart energetic state and removal of myocardial ischemia [24]. Furthermore, there is a dose response to Ad/*SERCA2a* transfer as overexpression of the *SERCA2a* gene promotes an additive increase in SERCA2a activity which further influences improved ventricular contractility [25] but has no effect on the atrium [26]. In addition to improvements in left ventricular contractility, Ad/*SERCA2a* gene transfer has also been shown to promote reverse myocardial remodeling by reducing left ventricular anterior wall thickening in Wistar rats with CHF, which reduced arrhythmic events and caused the restoration of left ventricular function similar to the observation in control animals [27].

The benefits of *SERCA2a* gene transfer via Ad-based vectors were not only limited to the myocardial tissue, however, as it also showed benefits on the vascular smooth muscle cells by preventing vascular remodeling and inhibition of neointimal thickening in a human ex vivo model of the coronary artery [28] and augmented coronary blood flow [29]. Although the Ad-based vector technique was successful overall, there were some inherent limitations to this gene delivery method, such as the presence of neutralizing antibodies and the severe immune response against Ad vector and/or the gene-modified cells [21]. Therefore, the development of more sophisticated viral vectors techniques was explored to overcome these limitations.

### 2.3. Adeno-Associated Virus (AAV)-Based Vectors

The AAV-based vector has significant advantage over Ad-based vector in gene delivery. To date, several generations of AAV systems have been adapted and adopted in the *SERCA2a* gene delivery with each generation improving delivery efficiency and safety over the previous one. AAV-based vector has been shown to safely and successfully alter gene expression in cardiac tissue [21], with much less inflammation than that associated with the Ad-based vector technique [21]. AAVs comprise more than 100 serotypes and each serotype presents distinct transduction efficiency in different tissues [21]. Among these AAVs, the AAV-1 was the best studied and AAV1-based vector is most commonly used. Indeed, several earlier trials were conducted using the AAV1/SERCA2a intra-coronary injection method in animal CHF models with good success. Hadri and colleagues demonstrated that long-term SERCA2a overexpression via in vivo AAV1-mediated gene transfer improves left ventricular ejection fraction, coronary artery blood flow, and expression of endothelial nitric oxide synthase in a swine model of CHF [30]. Similarly, AAV1/*SERCA2a* gene delivery improves echocardiography derived myocardial function and decreases myocardial apoptosis in pigs [31], as well as restores SERCA2a activity and protein expression following atrial fibrillation-related declines in SERCA2a activity and expression [32]. Finally, *SERCA2a* gene transfer not only effects cardiac tissue but also directly influences vascular endothelial and smooth muscle cell function by improving Ca^2+^ signaling [33] which highlights SERCA2a as an important therapeutic target for treating cardiovascular disease—including CHF.

Overall, AAV1 and AAV6 mediated-*SERCA2a* gene delivery methods have provided beneficial improvements in myocardial and coronary artery Ca^2+^ handling, contributing to improved cardiac function and reverse remodeling in animal models of CHF. This initial success in improving cardiac function in animal models led to large-scale clinical trials in humans which will be discussed in the following section.

## 3. Human Trials

Over the past decade several large-scale clinical trials have been performed using AAV1/SERCA2a in the treatment of human CHF (Table 1) and are discussed below.

### 3.1. The Calcium Upregulation by Percutaneous Administration of Gene Therapy in Cardiac Disease (CUPID) Trial

In response to the promising preclinical models in animals that demonstrated AAV1/SERCA2a were well tolerated and improved cardiac function, CUPID was the first-in-human clinical trial to evaluate the efficacy of recombinant AAV1/SERCA2a in human CHF [12,13]. The initial CUPID multicenter trial included two phases, wherein phase 1 was an open-label dose escalation protocol and phase 2, a randomized, double-blind, placebo-controlled trial. The overall primary outcome of the CUPID trials was to monitor safety, while secondary outcomes included improvements in physical activity and efficacy.

In CUPID phase 1, 12 patients were administered single intracoronary infusions, comparing 3 dose levels of AAV1/SERCA2a (AAV1 capsid with human SERCA2a cDNA flanked with inverted terminal repeats) with placebo, and primary outcomes were measured at 6 and 12 months post-infusion. Patients were enrolled independent of CHF etiology, were NYHA class III/IV, and had exhausted all current pharmacologic therapy. The results of phase 1 suggested that intracoronary infusion of AAV1/SERCA2a demonstrated an acceptable safety profile given the advanced CHF population. Several of the patients exhibited improvements in CHF symptoms and left ventricular structure and function at 6 months follow-up [12]. Of note, two of the patients who did not respond to therapy already had pre-existing neutralizing antibodies (NAb) for the viral capsid proteins. Pre-existing natural exposure to NAb, which are known to inhibit vector uptake, limit the efficacy of AAV1 treatment in CHF [34,35,36]. Discussion of the impact of NAb on vector uptake are beyond the scope of the current review and have been comprehensively described elsewhere [37].

The relative success of phase 1 led to initiation of phase 2, including 39 advanced CHF patients randomized to 3 doses of viral vector; low (6 × 10^11^ DNase resistant particles; DRP), medium (3 × 10^12^ DRP), and high doses (1 × 10^13^ DRP). Endpoints included patients’ symptomatic (NYHA functional class, Minnesota Living with Heart Failure Questionnaire; MLWHFQ) and functional (maximal oxygen uptake, 6-minute Walk Test) status, blood biomarker levels (N-Terminal-pro Brain Natriuretic Peptide, NT-proBNP), and left ventricular function and remodeling (Ejection fraction and End-systolic volume). One-year follow-up suggested the patients randomized to the “high” treatment dose of AAV1/SERCA2a improved in functional class (Mean reduction in MLWHFQ: −10.3 ± 12.21), blood biomarker (NT-proBNP mean reduction: 12.4 ± 47.83%) and left ventricular function (end-systolic volume mean reduction: −9.6 ± 27.55 mL; −4 ± 13.76%). Importantly, there was a significant increase in the time to cardiac event in all AAV1/SERCA2a groups in the CUPID trial, suggesting that perhaps even the lower doses of viral vector had beneficial effects on patient outcome, independent of physiological changes that are not appreciable at smaller sample sizes [13].

### 3.2. Post-CUPID

Since the conclusion of CUPID, three clinical trials utilizing AAV1 have targeted SERCA2a in human CHF [14,38,39,43]. CUPID2, a continuation of the CUPID clinical trials, was the first to recruit patients from outside the United States, enrolling a total of 250 patients with CHF. Patients were randomized to 10-minute intracoronary infusion of either placebo or “high” dose of AAV1/SERCA2a. In contrast to the results of the original CUPID trial, CUPID2 did not reduce either recurrent heart failure events, or terminal events in the study population [14,39].

The other two post-CUPID trails began in the United Kingdom, known as SERCA-LVAD and AGENT-HF. SERCA-LVAD aimed to take tissue biopsies at the time of left ventricular assist device implantation (pre-infusion of AAV1/SERCA2a) and at the time of transplant or LVAD removal (post-infusion) in order to correlate changes in clinical outcome with SERCA2a expression levels [43]. AGENT-HF utilized AAV1/SERCA2a with left ventricular structure and function as the primary outcome variables assessed at 6 months post-treatment [38]. Unfortunately, due to the negative results of the CUPID2 trial and lack of preliminary success, both studies were terminated prematurely. The studies showed no improvement in ventricular remodeling but were underpowered to demonstrate any effect of AAV1/*SERCA2a* gene therapy [38]. Taken together, the human trials utilizing AAV1/SERCA2a to treat HF have been widely unsuccessful to date.

The early clinical trial CUPID provided the basis for several more aggressive clinical trials (CUPID2, Agent-HF, SERCA-LVAD), all of which failed to demonstrate improvement in survival and/or were terminated prior to sufficient data collection. The reason for the failure of CUPID2 remains largely unknown. One possible reason is attributable to the insufficient delivery of SERCA2a DNA using intracoronary infusion of AAV1 [44], as intracoronary infusion may result in inadequate uptake of the viral vector into cardiomyocytes. In addition, work by Mingozzi and colleagues suggests that viral loads which include empty viral capsid particles enhance the gene delivery as they act as “decoys” and may block the inhibitory activity of antibodies [45]. Thus, *SERCA2a* gene therapy may not be at a dead end yet and there is some light through the tunnel to show it still as a valuable approach for HF patients. The challenge is rather how to effectively deliver the gene to cardiomyocytes. Are there better, more potent viral vectors and more advanced gene editing techniques for gene delivery and expression in human heart?

## 4. The Promise of Novel Gene Delivery Techniques in Animals

Among more than 100 wild-type AAV serotypes, some serotypes have been demonstrated to have distinct features on cardiomyocyte transduction [21]. Compared to AAV1, AAV8 showed 20-fold higher gene expression in the myocardium in mice. Systemic venous infusion of AAV9 triggers robust level (>200-fold) of gene expression in the cardiomyocytes when compared to AAV1 [46]. To date, AAV9-based vectors are by far the most efficient methods for delivering genes to cardiac tissue in mice [46,47,48]. However, AAV6 was also reported to mediate the most efficient transduction in mouse cardiac tissue [49].

Based on these findings, many recent studies have adopted the AAV9 method to transfer *SERCA2a* genes for treating CHF in animal models. Lyon et al. showed that *SERCA2a* gene transfer with AAV9 restored SERCA2a proteins in the rat heart and improved left ventricular function by reducing Ca^2+^ leakage from sarcoplasmic reticulum [50]. In addition, AAV9/SERCA2a therapy recovers the electrical signal constancy by improving ventricular arrhythmias [50,51] and the treatment rectifies ECG abnormalities such as tachycardia, shortened P-R interval, and prolonged Q-T intervals [52]. Similarly, *SERCA2a* gene therapy overexpressed SERCA2a protein and repressed atrial fibrillation induced by rapid pacing of the atrium in rabbit model [53]. Toward clinical application, this line of study has been moved forward to large animals as well. AAV9/*SERCA2a* gene therapies were performed in German pigs and ovine with ischemic CHF models [54]. AAV9 targeting the *S100A1* gene, which augments the activity of SERCA2a proteins so that Ca^2+^ signaling is improved, prevents left ventricular remodeling, and restores SERCA2a protein expression [54]. Likewise, injection of AAV9/SERCA2a into the coronary arteries significantly increased *SERCA2a* gene expression in the walls of the left ventricle in sheep [55]. This successful overexpression of SERCA2a triggered improvements in left ventricular systolic and diastolic function and subsequent left ventricular remodeling [55]. These positive outcomes provide the basis for future clinical trials in humans utilizing AAV9-based vectors. Current investigations in humans using AAV9-based vectors include Pompe disease (NCT02240407) and Batten disease (NCT02725580). Taken together, these data suggest that the AAV9/*SERCA2a* gene delivery is an appealing therapeutic method for targeting CHF due to its effects on the ventricular contractile function and restoration of electrical complications associated with the failing heart.

Besides AAVs, gene delivery method using other viral vectors is emerging. For example, lentiviral vectors have been demonstrated to be effective for SERCA2 gene transfer in rat ischemic HF model [56]. Since lentivirus can integrate the gene of interest into the host chromosome, this approach can achieve long-term effect of gene expression [57,58,59]. Many research groups have begun to examine safety and efficacy of this gene therapy approach in CHF animal models [56,60,61,62].

## 5. Concluding Remarks and Future Directions

The beneficial left ventricular functional and structural adaptations associated with AAV9/*SERCA2a* gene therapy provide solid preclinical evidence for future work to focus on translating these findings from animal work to human clinical applications. Compared to AAV1 which used in failed CUPID2 and other earlier trials, AAV9 presents superior efficiency and safety. Therefore, the improved Ca^2+^ handling in cardiac tissues in response to AAV9/SERCA2a would have significant benefits for patients suffering from CHF—particularly CHF of ischemic origin.

In addition to the focus on *SERCA2a* gene expression, there are a host of other molecules that both indirectly and directly modify SERCA2a function that are worthwhile investigating using modern gene therapy techniques. These molecules include, but are not limited to, small ubiquitin-related modifier-1 (SUMO-1) [63,64]. SUMO-1 is a post-transcriptional protein, which plays an important role in modifying other proteins involved in the preservation and stabilization of SERCA2a proteins. In porcine and human cardiac tissue, AAV1/SUMO-1 improved cardiac function. Concurrent transfection of both SUMO-1 and SERCA2a increased both expression and function of SERCA2a [63,64]. As such, this approach may represent a possible dual therapy in humans. Indeed, combined gene therapy has recently shown significant promise in the treatment of animal models of heart failure, with combined transfer of apelin, fibroblast growth factor-2, and SERCA2a demonstrating increased gene expression in ischemic heart failure model [61]. Therefore, future work should seek to translate the use of combined gene therapy in humans.

Moreover, novel pharmaceutical therapies targeting activation of SERCA2a have been developed. Istaroxime, a drug developed by Italian pharmaceutical company Sigma-Tau (Pomezia, Italy), is a lusitropic-inotropic drug recently approved for use in CHF [65]. In fact, currently a Phase 1 clinical trial at the University of Texas Southwestern is utilizing Istaroxime during exercise (NCT02772068), with primary outcome measure changes in cardiac filling pressures, and secondary outcome measure change in cardiac relaxation time. The study hopes to investigate the effect of SERCA2a activation on exercise-dependent changes in cardiac function (NCT02772068). At the same time, many other altered regulatory proteins involving in cardiomyocyte Ca^2+^ homeostasis could be potential targets for gene therapy in CHF as well, such as phospholamban, junctophilin-2 (JP2), RyRs and NCXs. For example, JP2 downregulation has been reported in human failing heart and restoring JP2 by AAV9-mediated gene therapy could rescue heart failure in mice [66,67,68,69].

In conclusion, the development of gene therapy and gene delivery methods have received significant progress over the past few decades, contributing to a novel therapeutic approach for treating CHF. Although the early promising AAV1/SERCA2a method in animal models failed to replicate the same results in humans, recent advancements in gene delivery techniques, especially AAV9/SERCA2a have provided compelling results for beneficial outcome of the upregulation of *SERCA2a* gene expression. Targeting SERCA2a for treatment of CHF in human patients should be still an option deserving further investigation. Future work should focus on translating the recent findings using AAV9/SERCA2a techniques into large-scale clinical trials in humans.

## Figures and Tables

**Table 1:** Current and previous clinical trials to test the efficacy of AAV1/SERCA2a on the Heart Failure.

National Clinical Trial Code	Status	Study Title	Conditions	Interventions	Study Results	References
NCT02772068	Recruiting	Hemodynamic Response to Exercise in HFpEF Patients After Upregulation of SERCA2a	Congestive Heart Failure	Drug: Istaroxime Other: Exercise	No Published Results	-
NCT01966887	Terminated	AAV1-CMV-Serca2a GENe Therapy Trial in Heart Failure (AGENT-HF)	Congestive Heart Failure Ischemic and non-ischemic Cardiomyopathies	Genetic: AAV1/SERCA2a (MYDICAR)-single intracoronary infusion Genetic: Placebo; single intracoronary infusion	No Positive or Negative Effects	[38]
NCT00534703	Terminated	Investigation of the Safety and Feasibility of AAV1/SERCA2a Gene Transfer in Patients with Chronic Heart Failure (SERCA-LVAD)	Chronic Heart Failure Left Ventricular Assist Device	Genetic: AAV1/SERCA2a Drug: Placebo	Terminated Early—No Results	-
NCT01643330	Completed	A Study of Genetically Targeted Enzyme Replacement Therapy for Advanced Heart Failure (CUPID-2b)	Ischemic and non-ischemic Cardiomyopathies Heart Failure	Genetic: AAV1/SERCA2a (MYDICAR) Genetic: Placebo	No Positive or Negative Effects	[12,13,14,37,39,40,41]
NCT00454818	Completed	Efficacy and Safety Study of Genetically Targeted Enzyme Replacement Therapy for Advanced Heart Failure (CUPID)	Heart Failure, Congestive Dilated Cardiomyopathy	Genetic: MYDICAR Phase 1 (Open-label, Serial Dose-Escalation Study) Procedure: Placebo Infusion Genetic: MYDICAR Phase 2 (Placebo-controlled, Randomized Study)	Positive Results	[12,13,40,41,42]
NCT02346422	Terminated	A Phase 1/2 Study of High-Dose Genetically Targeted Enzyme Replacement Therapy for Advanced Heart Failure	Heart Failure Cardiomyopathy	Genetic: MYICAR Phase 1/2 (Dose 2.5 × 1013 DRP)	Terminated Early—No Results	[12,13,40]
